# Generalized Confidence Intervals and Fiducial Intervals for Some Epidemiological Measures

**DOI:** 10.3390/ijerph13060605

**Published:** 2016-06-18

**Authors:** Ionut Bebu, George Luta, Thomas Mathew, Brian K. Agan

**Affiliations:** 1 The Biostatistics Center, Department of Epidemiology and Biostatistics, The George Washington University, 6110 Executive Blvd., Rockville, MD 20852, USA; 2 Department of Biostatistics, Bioinformatics and Biomathematics, Georgetown University, 4000 Reservoir Road, Washington, DC 20057, USA; George.Luta@georgetown.edu; 3 Department of Mathematics and Statistics, University of Maryland Baltimore County, 1000 Hilltop Circle, Baltimore, MD 21250, USA; mathew@umbc.edu; 4 Infectious Disease Clinical Research Program, Department of Preventive Medicine and Biometrics, Uniformed Services University of the Health Sciences, 4301 Jones Bridge Road, Bethesda, MD 20814, USA; bagan@idcrp.org

**Keywords:** common odds ratio, generalized pivotal quantity, fiducial quantity, log-binomial model, logistic regression

## Abstract

For binary outcome data from epidemiological studies, this article investigates the interval estimation of several measures of interest in the absence or presence of categorical covariates. When covariates are present, the logistic regression model as well as the log-binomial model are investigated. The measures considered include the common odds ratio (OR) from several studies, the number needed to treat (NNT), and the prevalence ratio. For each parameter, confidence intervals are constructed using the concepts of generalized pivotal quantities and fiducial quantities. Numerical results show that the confidence intervals so obtained exhibit satisfactory performance in terms of maintaining the coverage probabilities even when the sample sizes are not large. An appealing feature of the proposed solutions is that they are not based on maximization of the likelihood, and hence are free from convergence issues associated with the numerical calculation of the maximum likelihood estimators, especially in the context of the log-binomial model. The results are illustrated with a number of examples. The overall conclusion is that the proposed methodologies based on generalized pivotal quantities and fiducial quantities provide an accurate and unified approach for the interval estimation of the various epidemiological measures in the context of binary outcome data with or without covariates.

## 1. Introduction

This articles investigates inferences for several epidemiological measures of practical interest, in the absence or presence of covariates. In the latter scenario, both the logistic regression model and the log-binomial model will be considered. The logistic regression model plays a crucial role in the analysis of binary data arising from clinical trials and observational studies, and the focus of inferences is very often the odds ratio (OR). Another index that is very often used is the relative risk, or the risk ratio (RR). The RR measures the strength of association between a risk factor (or an exposure variable) and disease. Other related indices are the risk difference (RD), and the relative risk difference (RRD). The odds ratio computed under the logistic regression model is known to be a good approximation for the risk ratio for a rare outcome, but not so for an outcome that is common (*i.e.*, not rare). Another epidemiological measure of interest is the prevalence ratio (PR), which measures the association between prevalence of the health outcome and an exposure variable or risk factor. The log-binomial model can be used to estimate the risk ratio in the presence of covariates, when the outcome is not rare. Yet another measure of interest is the number needed to treat (NNT), which is the average number of patients needed to be treated to prevent an additional adverse outcome; the NNT is simply the reciprocal of the risk reduction. For randomized controlled trials with binary outcomes, the NNT is now widely used to measure the benefit of the treatment.

All of the above epidemiological measures are functions of the unknown parameters from the regression models, and inferences concerning them has been widely discussed in the literature, very often using standard likelihood based asymptotic methods [1,2,3,4]. We refer to these articles for background information and earlier literature on the point and interval estimation of the above epidemiological measures. The purpose of the present investigation is to explore the methodologies based on generalized confidence intervals and fiducial intervals for the interval estimation of the above quantities, and to assess their performance relative to the likelihood based large sample methods; performance in small sample scenarios will be of particular interest. The generalized confidence interval methodology is due to Weerahandi [5], and it has found numerous applications in interval estimation problems, resulting in confidence intervals that exhibit satisfactory performance in small samples; see also the books by Weerahandi [6,7]. In the context of binary data, the methodology was adopted to obtain satisfactory confidence intervals in a quantal assay problem [8] and in surrogate endpoint validation [9]. Recently, the fiducial approach has seen a revival; in fact, some of the generalized confidence intervals are indeed fiducial intervals. We refer to [10,11] for very detailed discussions of the fiducial methodology. It is important to notice that we are using all these intervals as confidence intervals in the usual frequentist sense, since it has been shown that they provide asymptotically correct frequentist coverage and have very good small sample properties.

We give a brief description of the generalized confidence intervals and fiducial intervals in the next section, and then explain their application for computing confidence intervals for the above epidemiological measures under the usual binomial model when covariates are absent, and under the logistic and log-binomial models when covariates are present. The fiducial approach was used for inferences concerning several parameters in the context of the binomial distribution (and also the Poisson distribution) in the absence of covariates [12]. As will become clear, the confidence intervals that we have derived do not rely on the maximum likelihood estimators, and hence are free of the computational issues associated with the maximization of the likelihood. This is of particular interest in the log-binomial model, since it is known that the restricted parameter space under the log-binomial model presents numerical difficulties, and the models may fail to converge while maximizing the likelihood [13,14]. Our proposed methodology based on generalized confidence intervals and fiducial intervals can also be used for stratified studies, when one is interested in constructing a confidence interval for the (assumed) common OR, for example.

As will be seen, the confidence intervals that we have constructed for the above interval estimation problems are conceptually simple and straightforward in terms of implementation. The performance of the proposed confidence intervals are assessed based on simulations, and illustrated using several examples. In terms of maintaining the coverage probabilities, the proposed confidence intervals turn out to be quite satisfactory, regardless of the sample size. Our overall conclusion is that the generalized confidence interval approach and the fiducial approach have resulted in a unified methodology for the interval estimation of various epidemiological measures, and the resulting confidence intervals exhibit satisfactory performance and are preferable to the likelihood based methods available in the literature.

## 2. Generalized Pivotal Quantities and Fiducial Quantities

The computation of a generalized confidence interval is based on the concept of a *generalized pivotal quantity* (GPQ). Similarly, the computation of a fiducial interval (FI) is based on a fiducial quantity. In this section, we define these. The GPQ and the fiducial quality are first introduced for a binomial setup without covariates (Section 2.1 and Section 2.2); they are then used to derive the corresponding quantities for the logistic regression model and the log-binomial model with categorical covariates.

### 2.1. Generalized Pivotal Quantity (GPQ)

In order to define a GPQ, let $X=(X_{1},X_{2},...,X_{n})$ be a random sample from a distribution that depends on a parameter of interest *θ*, and a nuisance parameter *δ*. Let *x* denote the observed value of *X*. A GPQ for *θ* is a function of *X*, *x*, *θ* and *δ*, say T(X,x;θ,δ), satisfying the following two conditions:

(i) Given the observed value *x*, the distribution of T(X,x;θ,δ) is free of any unknown parameter;
(ii) The observed value of T(X,x;θ,δ), *i.e.*, T(x,x;θ,δ), is free of the nuisance parameter *δ*.

We note that when T(X,x;θ,δ) is a GPQ for *θ*, as defined above, then for any scalar valued function h(θ) of *θ*, a GPQ is given by h(T(X,x;θ,δ)), and the percentiles of h(T(X,x;θ,δ)) can be used to obtain confidence limits for h(θ). The resulting confidence intervals are referred to as *generalized confidence intervals*. Sometimes, the distributional property (i) given above will hold only approximately; in this case, we will get only an “approximate GPQ”. This is indeed the situation for the problems investigated in this article. In what follows, we will refer to these approximate GPQs simply as GPQs.

The starting point for the computation of generalized confidence intervals for the various epidemiological measures we have considered is based on an approximate GPQ for the binomial parameter [8,9], and is obtained as follows. For a binomial distribution with parameter *p* and sample size *n*, our approximate GPQ for *p* is based on the normal approximation:

$$2\sqrt{n}\left(\arcsin\sqrt{\hat{p}}-\arcsin\sqrt{p}\right)∼N\left(0,1\right),$$

where $\hat{p}$ is the sample proportion. If ${\hat{p}}_{\mathrm{obs}}$ denotes the observed value of $\hat{p}$, a GPQ for *p* is given by,

(1) $$\begin{array}{ccc} {\tilde{T}}_{p} & = & {\left[\sin\left(\arcsin\sqrt{{\hat{p}}_{\mathrm{obs}}}-\frac{1}{2\sqrt{n}}2\sqrt{n}\left\{\arcsin\sqrt{\hat{p}}-\arcsin\sqrt{p}\right\}\right)\right]}^{2}\\ & = & {\left[\sin\left(\arcsin\sqrt{{\hat{p}}_{\mathrm{obs}}}-\frac{1}{2\sqrt{n}}Z\right)\right]}^{2}, \end{array}$$

where *Z* is standard normal. Quantiles of ${\tilde{T}}_{p}$ can be used as confidence limits for *p*. We shall briefly explain the estimation of the required quantiles by simulation, since such a simulation will be necessary to compute confidence limits for the various parameters that we shall take up in later sections. The quantiles of ${\tilde{T}}_{p}$ can be estimated by proceeding as follows. Once data are available, compute the observed proportion ${\hat{p}}_{\mathrm{obs}}$. Now generate Z∼N(0,1) *M* times (*M* = 10,000, for example), say $Z_{i}$, *i* = 1, 2, ...., *M*, and let ${\tilde{T}}_{pi}={\left[\sin\left(\arcsin\sqrt{{\hat{p}}_{\mathrm{obs}}}-\frac{1}{2\sqrt{n}}Z_{i}\right)\right]}^{2}$, *i* = 1, 2, ...., *M*. The 95th percentile of the sequence ${\tilde{T}}_{pi}$ provides a 95% upper confidence limit for *p*.

However, we note that, with the above definition, ${\tilde{T}}_{1-p}\neq 1-{\tilde{T}}_{p}$. This undesirable feature can be taken care of by using the quantiles of a sequence obtained by concatenating ${\tilde{T}}_{p}$ and $1-{\tilde{T}}_{1-p}$. In what follows, we shall use this approach.

### 2.2. Fiducial Quantity

Here, we shall not provide a general treatment of fiducial quantities; we refer to [10] for a very detailed discussion. We shall now exhibit two fiducial quantities for the binomial success probability *p*; the first one being an approximate fiducial quantity. In what follows, we will refer to these approximate fiducial quantities simply as fiducial quantities.

Consider a binomial random variable *X* with sample size *n* and success probability *p*. An approximate fiducial quantity for *p*, say $F_{1}\left(p\right)$, is given by:

(2) $$F_{1}\left(p\right)=\mathrm{Beta}\left(x+\frac{1}{2},n-x+\frac{1}{2}\right),$$

where *x* denotes the observed value of *X*. Such a fiducial quantity was previously used for obtaining a confidence interval for *p* [12]. A second fiducial quantity, say $F_{2}\left(p\right)$, is given in [10]:

(3) $$F_{2}\left(p\right)=U_{\left(x\right)}+\left[U_{\left(x+1\right)}-U_{\left(x\right)}\right]W,$$

where *x* denotes the observed value of *X*, $U_{\left(r\right)}$ is the *r*th order statistic based on a sample of size *n* from a uniform (0, 1) distribution, and *W* follows a uniform (0, 1) distribution, independent of $U_{\left(x\right)}$ and $U_{\left(x+1\right)}$, where x=0,…n, $U_{\left(0\right)}=0$, and $U_{\left(n+1\right)}=1$. An efficient algorithm to generate these order statistics is described in the Appendix A. The quantiles of the fiducial quantities can be estimated by proceeding as in the case of the GPQ, mentioned in the previous sub-section.

### 2.3. GPQs and Fiducial Quantities under the Logistic Regression Model

Since inferences concerning the various epidemiological measures under the logistic regression model will be taken up in this article, we shall now exhibit GPQs and fiducial quantities for the logistic parameters. Thus, consider Bernoulli responses where the success probability depends on categorical covariates through the logistic regression model, and suppose we have data corresponding to *m* covariate vectors, say $x_{i}$, *i* = 1, 2, ...., *m*, corresponding to the combinations of the values of the covariates. If $p_{i}$ denotes the probability of a positive response at the covariate vector $x_{i}$, we thus have

$$\mathrm{logit}\left(p_{i}\right)=x_{i}^{\prime }\beta$$

i=1,2,....,m, where β is a vector of unknown parameters. Suppose there are $n_{i}$ responses corresponding to the covariate vector $x_{i}$, and among these, let ${\hat{p}}_{i}$ denote the sample proportion of positive responses. We note that $n_{i}$ could be equal to one, and ${\hat{p}}_{i}$ is not available in this case. If $n_{i}>1$, so that ${\hat{p}}_{i}$ is available, let $T_{pi}$ denote the GPQ of $p_{i}$, as given in Equation (1). Consequently, $\mathrm{logit}(T_{pi})$ is a GPQ for $x_{i}^{\prime }\beta$. If $\tilde{T}$ is the vector consisting of the $\mathrm{logit}(T_{pi})$s, then $\tilde{T}$ is a GPQ for Xβ. Using this observation, we construct the following two GPQs for the vector β, denoted by $T_{1\beta }$ and $T_{2\beta }$:

(4) $$T_{1\beta }={\left(X^{\prime }X\right)}^{-1}X^{\prime }\tilde{T},\;T_{2\beta }={\left(X^{\prime }V^{-1}X\right)}^{-1}X^{\prime }V^{-1}\tilde{T},$$

where $X={\left(x_{1},x_{2},....,x_{m}\right)}^{\prime }$, and *V* is a diagonal matrix whose *i*th diagonal element is an approximate variance of $\mathrm{logit}(T_{pi})$. Using the delta method, an approximate variance is given by $1/\left(n_{i}\cdot {\hat{p}}_{i}\right)+1/\left(n_{i}\cdot \left(1-{\hat{p}}_{i}\right)\right)$.

Different fiducial quantities for β can be similarly constructed using $F_{1}\left(p\right)$ and $F_{2}\left(p\right)$ given in Equations (2) and (3). We note that in order to be able to construct the GPQs $T_{1\beta }$ and $T_{2\beta }$ given in Equation (4), we require each $n_{i}$ to be larger than one, where $n_{i}$ is the number of Bernoulli responses corresponding to the covariate vector $x_{i}$. However, in order to construct the fiducial quantities for β, some (or all) $n_{i}$s can be equal to one. While this is possible in principle, we noted that the performance of the resulting confidence intervals is not satisfactory, in terms of maintaining the coverage probability.

### 2.4. GPQs and Fiducial Quantities under the Log-Binomial Model

Under the log-binomial model, the probability $p_{i}$ for a positive response at the covariate vector $x_{i}$ is given by:

$$\ln\left(p_{i}\right)=x_{i}^{\prime }\beta$$

i=1,2,....,m, where β is a vector of unknown parameters. GPQs and fiducial quantities can now be constructed similar to what is given above for the logistic case; simply replace the logit function with the natural logarithm.

## 3. Generalized Confidence Intervals and Fiducial Intervals

Once a GPQ or a fiducial quantity is available for a parameter of interest, confidence limits can be obtained using the percentiles of the GPQ (or the fiducial quantity). This is precisely what is done in this section for the various epidemiological measures mentioned earlier. A property that we shall use is that if independent GPQs (or fiducial quantities) are available for several parameters, then a GPQ (or a fiducial quantity) for any function of the parameters can be obtained as the corresponding function of the GPQs (respectively, fiducial quantities). We start by considering the case of no covariates.

### 3.1. The Odds Ratio

We now apply the GPQ methodology and the fiducial approach for computing confidence intervals for the odds ratio from a single contingency table, or for the common odds ratio from several independent contingency tables. The case of a single contingency table has been addressed using the fiducial solution [12].

Consider two independent binomial random variables $X_{1}$ and $X_{2}$ with respective success probabilities $p_{1}$ and $p_{2}$, and respective sample sizes $n_{1}$ and $n_{2}$. Let ${\hat{p}}_{1}$ and ${\hat{p}}_{2}$ denote the sample proportions. The odds ratio is then defined as $\frac{p_{1}/\left(1-p_{1}\right)}{p_{2}/\left(1-p_{2}\right)}$. In the absence of covariates, an approximate GPQ for the odds ratio can be easily constructed, and is given by $\frac{T_{p_{1}}/\left(1-T_{p_{1}}\right)}{T_{p_{2}}/\left(1-T_{p_{2}}\right)}$, where $T_{p_{1}}$ and $T_{p_{2}}$ are defined similar to $T_{p}$ in Equation (1). Fiducial quantities can be similarly defined for the odds ratio. Percentiles of the quantities so obtained provide confidence intervals for the odds ratio. For example, the 2.5th and 97.5th percentiles of $\frac{T_{p_{1}}/\left(1-T_{p_{1}}\right)}{T_{p_{2}}/\left(1-T_{p_{2}}\right)}$ give 95% confidence limits for the odds ratio in the absence of covariates.

### 3.2. The Common Odds Ratio

Consider *K* independent studies (or strata from the same study), where from the *k*th study, we have observations for two independent binomial random variables $X_{1k}$ and $X_{2k}$ with respective success probabilities $p_{1k}$ and $p_{2k}$, and respective sample sizes $n_{1k}$ and $n_{2k}$, *k* = 1, 2, ...., *K*. Thus, the odds ratio from the *k*th study is $\delta_{k}=\frac{p_{1k}/\left(1-p_{1k}\right)}{p_{2k}/\left(1-p_{2k}\right)}$, *k* = 1, 2, ...., *K*. Assuming that the odds ratio is the same across the *K* studies, we have $\delta_{1}=\delta_{2}=....=\delta_{K}=\delta$ (say).

#### 3.2.1. An Approximate GPQ for the Common Odds Ratio

An approximate GPQ for each $\delta_{k}$, to be denoted by $T_{\delta_{k}}$, can be constructed from the *k*th study, proceeding as mentioned in Section 3.1. We now combine these GPQs in order to obtain an approximate GPQ for the common odds ratio *δ*. For this, we propose a weighted average of the study-specific GPQs on the log scale. The weights that we shall use are motivated as follows. For *i* = 1, 2, if ${\hat{p}}_{ik}$ denote sample proportions from the *k*th study, and if ${\hat{\delta }}_{k}=\frac{{\hat{p}}_{1k}/\left(1-{\hat{p}}_{1k}\right)}{{\hat{p}}_{2k}/\left(1-{\hat{p}}_{2k}\right)}$, *k* = 1, 2, ...., *K*, then using the delta method, an approximate variance of $\log({\hat{\delta }}_{k})$, say $1/w_{k}$, is given by:

$$1/w_{k}=\frac{1}{n_{1k}p_{1k}+0.5}+\frac{1}{n_{1k}\left(1-p_{1k}\right)+0.5}+\frac{1}{n_{2k}p_{2k}+0.5}+\frac{1}{n_{2k}\left(1-p_{2k}\right)+0.5},$$

where we have also used a continuity correction. Noting that $\log\left(\delta \right)=\sum_{k=1}^{K}w_{k}\log\left(\delta_{k}\right)/\sum_{k=1}^{K}w_{k}$, an approximate GPQ $T_{\delta }$ for the common odds ratio can be obtained from

$$\log\left(T_{\delta }\right)=\sum_{k=1}^{K}T_{w_{k}}\log\left(T_{\delta_{k}}\right)/\sum_{k=1}^{K}T_{w_{k}},\;\;$$

where

$$\begin{array}{ccc} T_{w_{k}} & = & \left\{\frac{1}{n_{1k}T_{p_{1k}}+0.5}+\frac{1}{n_{1k}\left(1-T_{p_{1k}}\right)+0.5}\right.\\ & & +\;\;\;{\left.\frac{1}{n_{2k}T_{p_{2k}}+0.5}+\frac{1}{n_{2k}\left(1-T_{p_{2k}}\right)+0.5}\right\}}^{-1}.\; \end{array}$$

The percentiles of $T_{\delta }$ can be used to obtain confidence intervals for the common odds ratio *δ*. Note that we have used data dependent weights that are meant to reflect the variability of each study-specific GPQ on the log scale. This is similar to the adaptive weights proposed in [15] in the context of robust meta-analysis using confidence distributions; see also [16], Section 6. Clearly, different choices are possible for the weights, as noted in [15,16], Section 6. Here, we have not investigated a comparison of the different choices for the weights. It is important to note that the approach described above for the common odds ratio can be easily extended to other measures such as the prevalence ratio and the relative risk.

#### 3.2.2. Fiducial Quantities for the Odds Ratio and the Common Odds Ratio

The procedures outlined above for constructing approximate GPQs for the odds ratio and the common odds ratio can easily be adapted for obtaining fiducial quantities for these parameters. In fact, we can obtain two fiducial quantities for each parameter, using Equations (2) and (3). The required derivations should be obvious and the details are omitted. It should be noted that for inferences concerning the odds ratio from a single study, a fiducial solution based on Equation (2) has been previously investigated [12].

### 3.3. Relative Risk and the Number Needed to Treat

It should be clear that proceeding along the lines of what has been done for the odds ratio and the common odds ratio, GPQs and fiducial quantities can be constructed for any scalar valued function of independent binomial parameters. In particular, if we have two independent binomial random variables $X_{1}$ and $X_{2}$ with respective success probabilities, $p_{1}$ and $p_{2}$, and respective sample sizes, $n_{1}$ and $n_{2}$, the relative risk is given by $p_{1}/p_{2}$, for which an approximate GPQ is given by $T_{p_{1}}/T_{p_{2}}$ (the notations are as before). Fiducial quantities can be similarly obtained. If $p_{1}$ and $p_{2}$ are the probabilities corresponding to an adverse event in a treatment group and a control group, respectively, then the number needed to treat (NNT) is given by $1/(p_{2}-p_{1})$. Thus, a confidence interval for the NNT can be obtained from a confidence interval for $p_{2}-p_{1}$. An approximate GPQ as well as fiducial quantities can be used for computing confidence intervals for $p_{2}-p_{1}$. In fact, fiducial quantities for these parameters based on Equation (2) are given in [12].

### 3.4. Epidemiological Measures under the Logistic and Log-Binomial Models

So far, our adaptation of the methodology based on GPQs and fiducial quantities has been for situations where covariates are absent. Clearly, the odds ratio, as well as the other epidemiological measures, have extensive practical applications in the context of binomial responses that depend on covariates. The logistic model is very often used to model the response probability. The log-binomial model is sometimes used to estimate the risk ratio in the presence of covariates, when the outcome is not rare. As noted in Section 2.4, under the log-binomial model, the binomial success probabilities *p* satisfies $\ln\left(p\right)=x^{\prime }\beta$, where x is a covariate vector. Writing $x={\left(x_{1},x_{2},....,x_{s}\right)}^{\prime }$ and $\beta ={\left(\beta_{1},\beta_{2},....,\beta_{s}\right)}^{\prime }$, where *s* is the number of covariates, the parameter $\beta_{1}$ is the prevalence ratio (PR) for a one unit increase in $x_{1}$, adjusted for the other covariates. We recall that GPQs and fiducial quantities for β are given in Section 2.3 and Section 2.4 for the logistic model and the log-binomial model, respectively. From this, GPQs and fiducial quantities can be constructed for any function of β; in particular, for the various epidemiological measures, including the prevalence ratio.

## 4. Numerical Results

The accuracy of the proposed procedures based on GPQs and fiducial quantities is assessed using simulations. Here, we have presented the results for only two scenarios: interval estimation of a common odds ratio (under binomial distributions without covariates), and the interval estimation of a prevalence ratio (under the log-binomial model). We refer to [12] for numerical results on the performance of fiducial intervals for a few other parameters, including that for the difference between binomial proportions. Note that coverage probability for the latter is equivalent to that for the NNT.

### 4.1. Common Odds Ratio

Table 1 gives the coverage probabilities of the confidence intervals based on different approaches for a common odds ratio from *K* = 5 studies, for a 95% nominal level. We also assume that, for the different studies, $n_{1k}=n_{1}$, and $n_{2k}=n_{2}$ (*k* = 1, 2, …, 5), where we have used the notations in Section 3.2. The following confidence intervals are considered for the comparison: (i) confidence interval based on the Mantel–Haentzel estimator (denoted by MH in Table 1); see [17] for details; (ii) the Sato–Mantel–Haentzel confidence interval (denoted by SMH in Table 1); (iii) confidence interval based on the GPQ (denoted by GPQ in the table); (iv) confidence interval based on the fiducial quantity Equation (2) (denoted by F1 in the table); and (v) confidence interval based on the fiducial quantity Equation (3), denoted by F2 in the table; the computation of these intervals is explained in Section 3.2.2. The notation OR in the table refers to the true value of the common odds ratio. We have also computed the mean length and the median length of the different confidence intervals (given within brackets in Table 1). In terms of coverage probability and expected mean length (or median length), the confidence interval based on the fiducial quantity Equation (2) appears to perform better than the other approaches in the simulation setups considered. The mean length as well as the median length of the interval based on Equation (2) is substantially lower compared to those based on MH, SMH and GPQ, while satisfactorily maintaining the coverage probability. While the interval based on Equation (3) exhibits comparable performance in many cases, its coverage probability is not as satisfactory as that of the interval based on Equation (2). The satisfactory performance of the fiducial approach for inferences concerning the odds ratio from a single study was previously noted [12].

### 4.2. A Log-Binomial Model

The simulation set up used here is motivated by Example 1 in [18]. Here, apart from a treatment indicator, we have gender as a covariate. Suppose $n_{1}$ male patients are assigned to the treatment, and $n_{2}$ to a placebo, and let $n_{3}$ and $n_{4}$, respectively, be the corresponding sample sizes for the females. With Bernoulli outcomes for each patient, we assume a log-binomial model for the probability of a positive response. Thus, if $p_{i}$ is the probability of a positive response, we assume the model $\ln\left(p_{i}\right)=\beta_{0}+\beta_{1}x_{1}+\beta_{2}x_{2}$, where the *β*’s are unknown parameters, $x_{1}$ is a binary indicator for the treatment, and $x_{2}$ is a binary indicator for gender. Then, exp($\beta_{1}$) is the prevalence ratio of interest. For various sample sizes and parameter choices, Table 2 gives the coverage probabilities of confidence intervals for $\beta_{1}$ using the GPQ, using the two fiducial quantities, and using the asymptotic normality of the maximum likelihood estimator (denoted by ML in the table). The mean lengths and median lengths are also given (the numbers within parenthesis in Table 2). It appears that all the approaches perform well in terms of coverage probabilities; the minor differences noted among the mean lengths and median lengths among the GPQ-based and fiducial-based solutions are perhaps due to the minor differences among the coverage probabilities. We also note that in terms of median lengths, the ML solution has a slight edge over the other solutions. However, its mean length is unusually large in a few cases. This could be a reflection of the convergence problems while maximizing the likelihood; we suspect that the information matrix is becoming close to being singular, resulting in wide intervals. Note that the solutions based on the GPQ approach and the fiducial approach are both free of this drawback.

## 5. Examples

We present four examples in this section in order to illustrate our interval estimation methodologies, and for making comparisons with other available intervals.

### 5.1. NNT: Depression and Insomnia

This example is based on data from a cross-sectional study of sleep disturbances among HIV-infected persons in an investigation of the association between depression and insomnia [19]. Insomnia was assessed using the Pittsburgh Sleep Quality Index (PSQI) (with a global score greater than five taken as indication of insomnia). Depression was assessed using the Beck Depression Inventory (BDI). The problem is to estimate the NNT, or, more precisely, the number needed to expose (NNE). The data are reported in Table 3.

Among subjects with normal levels of depression (BDI ≤9), 36.6% have insomnia (56/(56 + 97)), while among subjects with at least a mild level of depression (BDI ≥10), 82.5% have insomnia (33/(33 + 7)).

Thus, the estimated NNE is 1/(0.825 – 0.366) = 2.18. This means that, on average, among approximately every two subjects with a level of depression mild or above, there will be one additional insomnia case relative to the normal group. Ninety-five percent confidence intervals for the NNE are reported in Table 4. We have also included the Wald–Yates and Agresti–Caffo intervals [20] for comparison.

We note that the intervals based on the GPQ, as well as those based on F1 and F2, are shorter compared to the other two intervals.

### 5.2. Common Odds Ratio: Viral Suppression

The U.S. Military HIV Natural History Study (NHS) is a prospective continuous enrollment cohort study of consenting military beneficiaries with HIV infection including active duty personnel, retirees, and dependents [21]. In this example, we consider the subjects on highly active antiretroviral therapy (HAART) with at least one viral load value (VL) in the first year. A subject is considered viral suppressed (VS) if the VL value at the last visit during the first year is below 400 copies/mL. The goal is to compare the odds of VS between African-American (AA) subjects and Caucasian (C) subjects. Analyses are stratified by enrollment site to accommodate for potential difference in treatment practices. A total of 1796 subjects (AA and C) started HAART after January 1st 1996 at one of three sites, and have at least one VL value during the first year. Table 5 presents the VS status (counts) stratified by race and site. The counts represent those that are virally suppressed (under the Y column) and those that are not suppressed (under the N column).

The *p*-value for the Breslow–Day Test [22] for homogeneity of the odds ratios across the sites is 0.48. Thus, we proceed under the assumption of a common odds ratio.

The 95 confidence intervals for the common odds ratio, based on the different methods are reported in Table 6.

### 5.3. Logistic Regression with Categorical Covariates: Treatment of AIDS

This example is taken from [23], and the data provide counts on the presence or absence of symptoms among AIDS patients who are on the antiretroviral drug AZT, categorized by race (White or Black). Thus, race is a binary covariate. The data are reported in Table 7.

Following [23], we assume a model without an interaction between race and treatment. If *p* denotes the proportion having symptoms, we model it as logit$\left(p\right)=\beta_{0}+\beta_{1}x_{1}+\beta_{2}x_{2}$, where $x_{1}$ is a binary covariate for race, and $x_{2}$ is a binary covariate that categorizes a patient as taking AZT or not taking it. Thus, if $p_{WY}$ denotes the proportion having symptoms among the whites who take AZT, and $p_{WN}$, $p_{BY}$ and $p_{BN}$ similarly defined, the model for (logit($p_{WY}$), logit($p_{WN}$), logit($p_{BY}$), logit($p_{BN}{))}^{\prime }$ can be written as:

$$\left(\begin{array}{ccc} 1 & 1 & 1\\ 1 & 1 & 0\\ 1 & 0 & 1\\ 1 & 0 & 0 \end{array}\right)\left(\begin{array}{c} \beta_{0}\\ \beta_{1}\\ \beta_{2} \end{array}\right),$$

similar to what is presented in Section 2.3. Based on the second GPQ in (4), we computed confidence intervals for $\beta_{1}$ and $\beta_{2}$, and they are given below. For comparison, we have also included the Wald interval. Results are similar: adjusted for race, treatment is effective in reducing the probability of developing AIDS symptoms (corresponding to $\beta_{2}$), while adjusted for treatment, there was no difference in outcome based on race (corresponding to $\beta_{1}$).

**Wald****GPQ****F1****F2**exp(*β*_1_)(0.600,1.861)(0.591,1.862)(0.600,1.860)(0.599,1.865)exp(*β*_2_)(0.282,0.841)(0.277,0.851)(0.281,0.849)(0.283,0.846)

### 5.4. A Log-Binomial Model: Migraine Headaches

We now revisit Example 1 in [18], a clinical trial for treatment of migraine headaches; some details of the example are presented in Section 4.2 and will not be repeated here. Here, we have the log-binomial model $\ln\left(p_{i}\right)=\beta_{0}+\beta_{1}x_{1}+\beta_{2}x_{2}$; we once again refer to Section 4.2 for an explanation of the notations. We shall consider the interval estimation of the prevalence ratio $\exp(\beta_{1})$. Using the data in [18], the maximum likelihood estimators (MLEs) of the *β*’s are ${\hat{\beta }}_{0}$ = −1.398, ${\hat{\beta }}_{1}$ = 0.783 and ${\hat{\beta }}_{2}$ = −0.151. Thus, we have the estimated prevalence ratio exp(0.783) = 2.189. The 95% confidence intervals for $\beta_{1}$, obtained by different methods are given in Table 8. We have also included the likelihood based interval.

We note that the intervals based on the different approaches are all very similar. This is also consistent with the numerical results in Table 2, since the different approaches (including the ML, when it converged) resulted in similar coverage probabilities and mean lengths (as well as the median lengths).

## 6. Discussion

Interval estimation of various epidemiological measures is of considerable practical significance while analyzing data from epidemiological studies. The present work addresses this problem for a variety of measures when we have binary outcomes. This investigation has been motivated by two practical considerations: accuracy of the confidence intervals in terms of maintaining the coverage probability close to the nominal level (especially in small samples), and ease of computation. The concepts of generalized pivotal quantities and fiducial quantities appear to provide confidence intervals that meet both of these requirements for a variety of epidemiological measures. In short, the approaches described here appear to provide a unified methodology for obtaining accurate and easy to use confidence intervals for binary data under the logistic regression model, and also under the log-binomial model. The computational advantage could be especially interesting in the context of the log-binomial model, since the model is known to present computational challenges (lack of convergence) while trying to compute the MLEs; this issue came up in the context of the numerical results in Table 2. A major advantage of the methodologies proposed here is that they are not based on the MLEs, and there is no need to compute the MLEs.

Our work can be extended in several directions. First, the generalized and the fiducial quantities proposed and investigated herein are frequentist in nature, and therefore only frequentist methods were considered. It would be of interest to further compare them with Bayesian approaches [24]. Second, other fiducial quantities can be considered. For example, a generalized fiducial quantity was obtained in [25] by solving a data-generating equation in the context of binary logistic item response models. The solution is not unique, but the impact of the selection rule is usually asymptotically negligible [26].

The log-binomial model imposes a natural constraint on β, namely, $x_{i}^{\prime }\beta \leq 0$ (using the notation in Section 2.4). Consequently, a GPQ (or a fiducial quantity) $T_{\beta }$ of β must satisfy $x_{i}^{\prime }T_{\beta }\leq 0$. The construction of the GPQ (as well as the fiducial quantity) described in this article is not guaranteed to meet this condition. One approach to have this constraint satisfied is to consider the projection of the GPQ onto the convex set defined by the constraint. Such a projection will also be a GPQ. However, this could present a methodology that is computationally demanding, and we have not pursued it in the present investigation.

## 7. Conclusions

The generalized confidence interval approach and the fiducial approach provide a unified methodology for the interval estimation of various epidemiological measures. The resulting confidence intervals exhibit satisfactory performance in terms of maintaining the coverage probability close to the nominal level.

## Figures and Tables

**Table 1:** Empirical coverage probability and (mean,median) length of different confidence intervals for the common odds ratio for five studies, for a 95% nominal level.

n1	n2	OR	MH		SMH		GPQ		F1		F2	
15	15	1.0	0.9520	(1.70,1.56)	0.9437	(1.69,1.55)	0.9489	(1.68,1.53)	0.9446	(1.58,1.45)	0.9467	(1.53,1.41)
15	15	3.5	0.9530	(7.24,6.12)	0.9533	(7.07,6.06)	0.9469	(7.79,6.34)	0.9507	(6.50,5.49)	0.9382	(5.94,5.14)
15	15	6.5	0.9580	(16.72,12.89)	0.9503	(16.65,12.83)	0.9526	(19.53,14.42)	0.9485	(15.22,11.84)	0.9352	(12.51,10.36)
20	10	1.0	0.9552	(1.85,1.69)	0.9490	(1.82,1.65)	0.9480	(1.83,1.64)	0.9464	(1.71,1.55)	0.9453	(1.63,1.50)
20	10	3.5	0.9556	(8.42,6.82)	0.9513	(7.96,6.50)	0.9511	(9.35,7.19)	0.9497	(7.29,5.99)	0.9401	(6.43 ,5.45)
20	10	6.5	0.9570	(22.04,14.80)	0.9521	(20.82,14.14)	0.9534	(25.00,17.23)	0.9500	(17.78,13.17)	0.9377	(13.79,11.06)
20	20	1.0	0.9527	(1.41,1.33)	0.9502	(1.39,1.31)	0.9510	(1.36,1.27)	0.9501	(1.31,1.23)	0.9502	(1.29,1.21)
20	20	3.5	0.9544	(5.73,5.14)	0.9516	(5.67 ,5.07)	0.9466	(5.78,5.03)	0.9474	(5.10,4.56)	0.9381	(4.87,4.40)
20	20	6.5	0.9541	(12.89,10.84)	0.9524	(12.59,10.66)	0.9469	(14.12,11.35)	0.9465	(11.34,9.59)	0.9363	(10.37,8.97)
30	30	1.0	0.9492	(1.10,1.06)	0.9511	(1.10,1.05)	0.9473	(1.06,1.02)	0.9512	(1.04,1.00)	0.9460	(1.03,0.99)
30	30	3.5	0.9506	(4.35,4.07)	0.9505	(4.32,4.01)	0.9480	(4.18,3.87)	0.9471	(3.89,3.63)	0.9425	(3.82,3.59)
30	30	6.5	0.9552	(9.38,8.49)	0.9506	(9.30,8.36)	0.9457	(9.39,8.28)	0.9419	(8.27,7.44)	0.9357	(7.94,7.22)

OR = odds ratio, MH = Mantel-Haentzel, SMH = Sato-Mantel-Haentzel, GPQ = generalized pivotal quantity, F1 = Equation (2), F2 = Equation (3).

**Table 2:** Empirical coverage probability and (mean,median) length of different confidence intervals for the prevalence ratio in the log-binomial model mentioned in Section 4.2, for a 95% nominal level.

*β*_0_	*β*_1_	*β*_2_	*n*_1_	*n*_2_	*n*_3_	*n*_4_	GPQ		F1		F2		ML	
–1.4	0.7	–0.2	28	28	26	26	0.9540	(1.34,1.27)	0.9438	(1.28,1.23)	0.9412	(1.26,1.21)	0.9526	(1.19,1.16)
–1.4	0.9	–0.2	28	28	26	26	0.9552	(1.28,1.22)	0.9445	(1.23,1.18)	0.9417	(1.21,1.16)	0.9534	(1.14,1.11)
–2.0	0.7	–0.2	28	28	26	26	0.9607	(2.22,2.01)	0.9419	(2.09,1.88)	0.9365	(1.97,1.83)	0.9652	(5.90,1.74)
–2.0	0.9	–0.2	28	28	26	26	0.9590	(2.15,1.94)	0.9361	(2.03,1.81)	0.9311	(1.91,1.77)	0.9653	(21.94,1.67)
–2.0	0.5	–0.2	28	28	26	26	0.9557	(2.35,2.12)	0.9357	(2.21,1.97)	0.9308	(2.07,1.93)	0.9640	(11.14,1.82)
–1.4	0.7	–0.2	56	56	52	52	0.9514	(0.87,0.86)	0.9452	(0.86,0.85)	0.9453	(0.85,0.84)	0.9496	(0.83,0.82)
–1.4	0.9	–0.2	56	56	52	52	0.9529	(0.84,0.82)	0.9475	(0.82,0.81)	0.9462	(0.82,0.81)	0.9516	(0.79,0.79)
–1.4	0.5	–0.2	56	56	52	52	0.9515	(0.91,0.90)	0.9464	(0.90,0.89)	0.9447	(0.90,0.88)	0.9506	(0.87,0.86)
–2.0	0.7	–0.2	56	56	52	52	0.9539	(1.36,1.30)	0.9447	(1.32,1.27)	0.9426	(1.30,1.26)	0.9568	(1.24,1.21)
–2.0	0.9	–0.2	56	56	52	52	0.9509	(1.31,1.25)	0.9429	(1.27,1.22)	0.9407	(1.25,1.21)	0.9540	(1.20,1.17)
–2.0	0.5	–0.2	56	56	52	52	0.9538	(1.42,1.37)	0.9454	(1.38,1.33)	0.9429	(1.36,1.32)	0.9583	(1.94,1.27)

GPQ = generalized pivotal quantity, F1 = Equation (2), F2 = Equation (3), ML = maximum likelihood.

**Table 3:** Cross-classification of depression and insomnia cases.

		Insomnia	
		No	Yes
**Depression**	**No**	97	56
	**Yes**	7	33

**Table 4:** 95% confidence intervals for the number needed to expose for the depression and insomnia example.

	Wald-Yates	Agresti-Caffo	GPQ	F1	F2
Lower limit	1.63	1.71	1.71	1.73	1.73
Upper limit	3.30	3.35	3.26	3.33	3.32

GPQ = generalized pivotal quantity, F1 = Equation (2), F2 = Equation (3).

**Table 5:** Number of subjects virally suppressed (Y) and not suppressed (N) stratified by race (AA = African-American, C = Caucasian) and site.

	Site1		Site2		Site3	
	Y	N	Y	N	Y	N
AA	212	90	11	2	352	272
C	271	108	17	3	293	165

**Table 6:** 95% confidence intervals for the common odds ratio for the viral suppression example.

	MH	SMH	GPQ	F1	F2
Lower limit	0.656	0.653	0.655	0.657	0.655
Upper limit	0.973	0.926	0.974	0.970	0.974

MH = Mantel-Haentzel, SMH = Sato-Mantel-Haentzel, GPQ = generalized pivotal quantity, F1 = Equation (2), F2 = Equation (3).

**Table 7:** Counts on the presence or absence of symptoms among AIDS patients who are on the antiretroviral drug AZT, categorized by race.

		Symptoms	
Race	AZT	Yes	No
White (W)	Yes (Y)	14	93
	No (N)	32	81
Black (B)	Yes (Y)	11	52
	No (N)	12	43

**Table 8:** 95% confidence intervals for the prevalence ratio in the migraine headaches example.

	ML	GPQ	F1	F2
Lower limit	0.223	0.221	0.233	0.245
Upper limit	1.344	1.412	1.395	1.379

ML = maximum likelihood, GPQ = generalized pivotal quantity, F1 = Equation (2), F2 = Equation (3).

## Appendix A. Order Statistics

Notice that for 0<x≤n, one has $U_{\left(x\right)}∼Beta\left(x,n-x+1\right)$. In addition, the joint distribution of $\left(U_{\left(x\right)},U_{\left(x+1\right)}\right)$ is given by:

$$f_{U_{\left(x\right)},U_{\left(x+1\right)}}\left(u,v\right)=\frac{n!}{(x-1)!(n-x-1)!}u^{x-1}{\left(1-v\right)}^{n-x-1},$$

and one can use a conditional approach to simulate from this distribution as follows:

1. Generate $U_{\left(x+1\right)}∼Beta\left(x+1,n-x\right)$;
2. Generate W∼Unif(0,1), *W* and $U_{\left(x+1\right)}$ are independent;
3. Compute $U_{\left(x\right)}=W^{1/x}U_{\left(x+1\right)}$.
